# Study of the hydrogeochemical processes and its impact on the quality of groundwater in the area between Abu Simbil and Tushka, Western Desert, Egypt

**DOI:** 10.1038/s41598-025-90669-x

**Published:** 2025-03-11

**Authors:** Saad A. Mohallel

**Affiliations:** Hydrogeochemistry Department, Desert Research Center, 1-Mathaf El Mataryia St., El Mataryia, Cairo, Egypt

**Keywords:** Groundwater, Hydrogechemical processes, Water quality, Western Desert, Mixing model, Environmental sciences, Chemistry

## Abstract

The study area is located in a hyper arid area in the Western Egyptian Desert, which represents a massive agricultural project where irrigation water is extracted from the Nubian sandstone aquifer. This study focuses on the hydrogeochemical processes and its impact on the quality of the groundwater aquifer. Based on the geomorphology, the study area includes five geomorphological units, Wadi Kurkur bediplain, Aswan High Dam Lake, the Nile Valley, the West Dungul plain, and basement outcrops. According to the geology, the study area is distinguished by sedimentary cover ranging in age from Upper Jurassic to Quaternary. Regarding the hydrogeology of the study area, the Nubian sandstone aquifer is the main aquifer in the study area, which it is represented by 24 groundwater samples plus one sample from the stem of the Lake Nasser. All samples were collected, analyzed, and interpreted. Groundwater salinity classification shows that all the groundwater samples are fresh water types, except one sample considered brackish water type. The pH values of the selected groundwater samples vary from 5.8 in the center to 7.6 in the northern side, with a median value of 7.3. The relationship between salinity content and the other ions shows a strong correlation between the values of Ca^2+^, Cl^−^, and SO_4_^2−^ with the TDS values and a moderate correlation between Na^+^ values with TDS values. Silicate and carbonate weathering are the main hydrochemical processes affecting the groundwater. From the saturation indices (SI) results, it is indicated that gypsum, anhydrite and manganite dissolve with negative SI values, whereas iron minerals are supersaturated with positive values. The hypothetical salts indicate a recharge from the lake Nasser in addition to leaching and dissolution of terrestrial salts. From the mixing Model, it is indicated that the Nubian sandstone aquifer has paleowater contribution percent ranging from 81 to 92% and Lake Nasser water contribution percent ranging from 7 to 18%. The nitrate concentrations are below the maximum allowable limits of the WHO; however, high concentrations of heavy metals were recorded in the groundwater samples at various extents.

## Introduction

In Egypt, the spatial distribution of water resources is imbalanced. About 96% of its land endures continuous droughts^1^, where 98% of the population lives on 4% of its land area. The majority of the Egyptian people live near the Nile River. Consequently, the river’s flow has historically meant the difference between life and death^2^. Studies of climate change impact on the Nile River show that the basin is extremely sensitive to temperature and precipitation changes^3^. An increase of 10% in average annual precipitation would lead to an average increase in annual flow of 40%. Similarly, a decrease in 10% in precipitation would lead to a reduction of the annual flow of more than 50%^4^. Also, another frontier condition is the Grand Ethiopian Renaissance Dam (GERD) on the Blue Nile^5,6^. This is considered the most critical challenge, posing the greatest threat to Egyptian water security. In addition, the increasing population in Egypt constitutes a big problem because of its great effect on the national growth, leading to an increase in poverty and social problems. The reclamation of new land for agriculture remains one of the major solutions to minimize such harmful effects. In Egypt, some governmental and agricultural programs are now under execution, while others are planned to start in the near future^7^. These programs depend on the surface water from Lake Nasser and the Nubian Sandstone groundwater. Due to the importance of the groundwater sources to the area adjacent to Lake Nasser for the development of the area. Reclamation of the study area has already begun, which encourages the monitoring of the hydrogeochemical processes induced in the aquifer and its impact on the quality of the groundwater in the study area, which needs to update especially with the expansion of agricultural areas. The area under investigation has been studied by many researchers, such^8^ who studied the hydrology of the study area and stated that there is a recharge from the Lake Nasser for about 50 km distance from the Lake Nasser border. In addition^9^, studied the hydrogeochemistry of the study area and concluded that surface water leakage into the Nubian sandstone aquifer occurs proportionally in all parts of the study area. Furthermore^7^ studied the statistical analysis of the hydrochemical data to identify the geochemical processes in the Nubian sandstone aquifer and concluded that the main productive aquifer in the study area is the Nubian sandstone aquifer and there are three statistical affected components. The three components of the PCA account for 75.83% of the total variance in the data set. Component 1is defined by highly positive loading explained in SO_4_^2−^, Ca^+^, Na^+^, Mg^2+^, and HCO_3_^−^ in addition to relatively high positive loading in Cl^−^, this is related to leaching and dissolution of terrestrial salts and some influences by agricultural activities in the cultivated areas. Components 2 and 3 are related to more local and geological effects. The present study aims to study the hydrogeochemical processes affecting on the groundwater for the future of development, through the study of the hydrogeochemical processes and their impacts on the quality of the groundwater. Furthermore, apply a mixing model to delineate the exact percent of the recharge from Lake Nasser in each well in the study area. In addition, our objective was to better identify the processes controlling the hydrogeochemical evolution of groundwater in the study area by using the hydrogeochemical criteria with different methods, because the water table in the study area is influenced by several factors, including evaporation, mixing, leaching and dissolution of different salts and agricultural return water flow.

## Study area

### Location

**Fig. 1:**
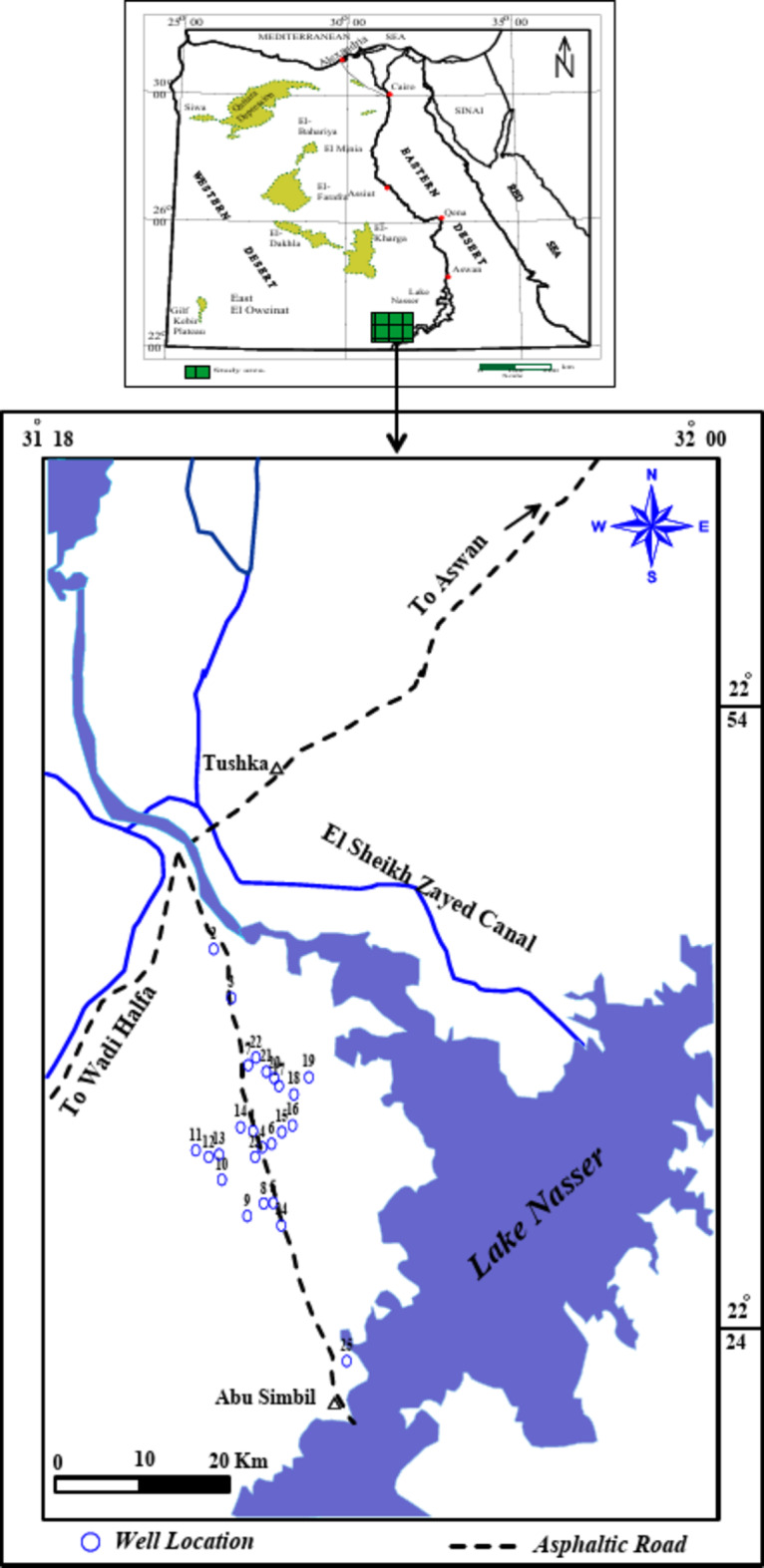
Wells’ location map of the study area.

The investigated area is located on the western side of Lake Nasser, where it occupies a surface area of about 11,000 km^2^ between latitudes 22° 24′ & 22° 50′ N and longitudes 31° 18′ & 32° 00′ E (Fig. 1).

The study area is located within the hyper-arid zone, where precipitation occurs once every two or three decades. Data obtained from the Tropical Rainfall Measuring Mission (TRMM) shows that the study area has received less than 20 mm of total rainfall from September 1999 to September 2006. This area is characterized by very hot summers and cold winters, and the air temperature ranges in summer between 49.5 C and 27.3 C and in winter between 30 C and 15 C, while relative humidity ranges between 43% and 18% in summer and between 43% and 10% in winter^10^.

### Geomorphology

**Fig. 2:**
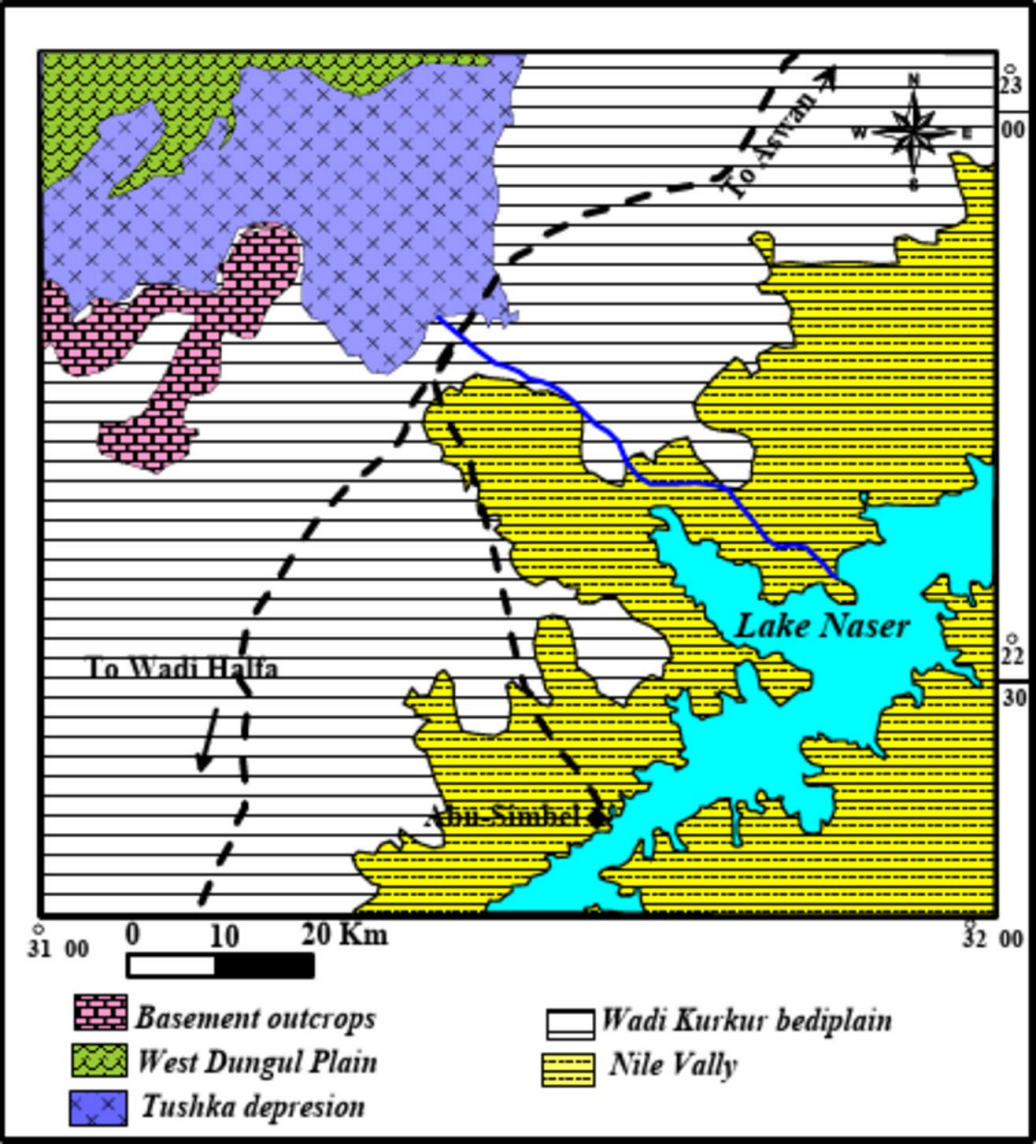
Geomorphologic map units Compiled after^11,12^.

According to^8,10–12^ five geomorphologic units could be distinguished in the study area (Fig. 2). Wadi Kurkur bediplain, Aswan High Dam Lake, The Nile Valley, West Dungul Plain, Tushka depression, and basement outcrops. Wadi Kurkur bediplain represents most parts of the area.

The bediplain surface is covered mainly by nearly horizontal beds of Nubian sandstone, with outcrops of igneous and metamorphic rocks as well as several volcanic exposures.

### Geology

**Fig. 3:**
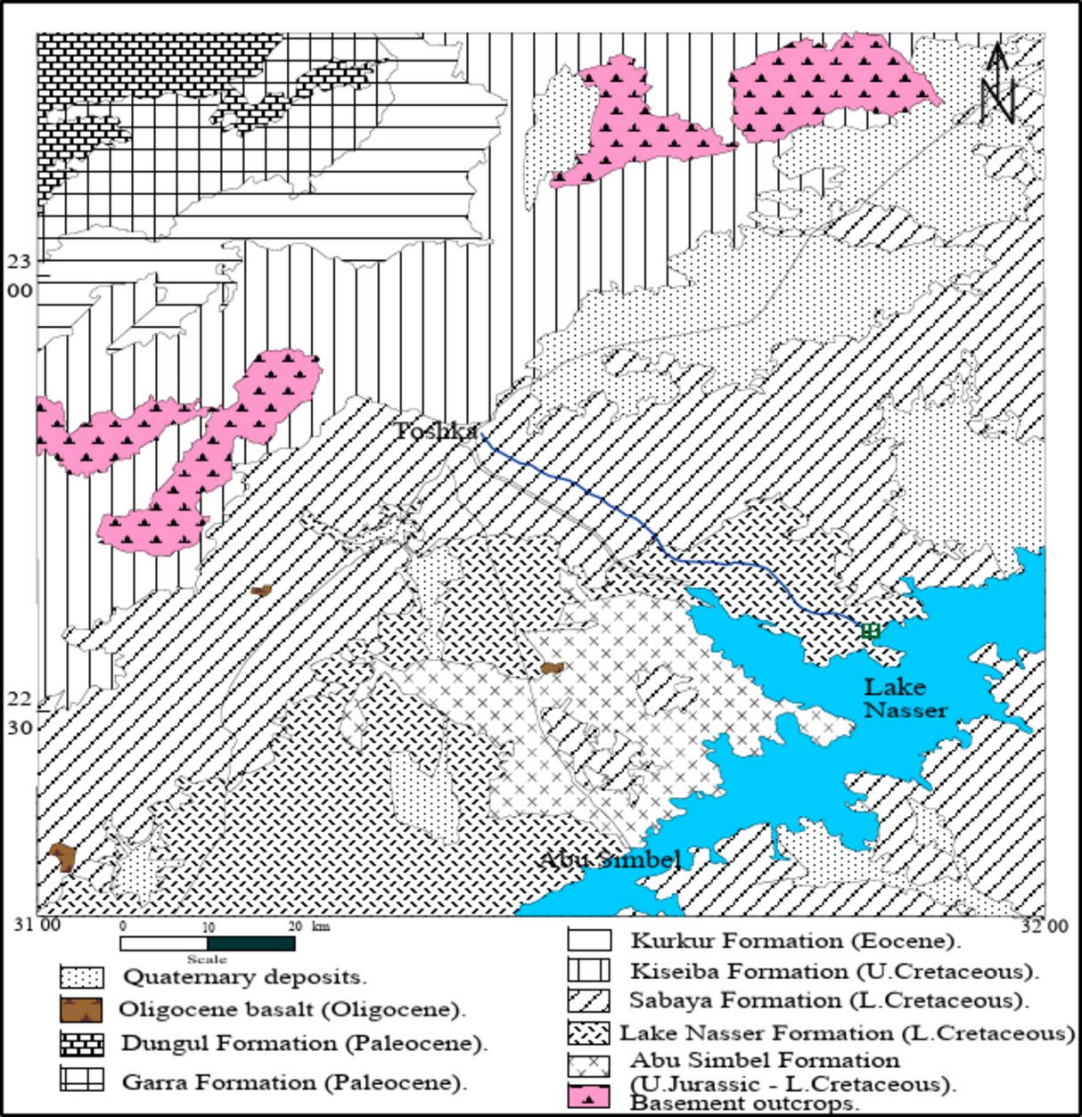
Geologic map after^12^.

Geologically, the investigated area is characterized by sedimentary cover ranging in age from the Upper Jurassic to recent^12^. The geological units are clarified from older to younger as follows (Fig. 3):

1. Upper Jurassic—Lower Cretaceous rocks (Abu Simbel Formation) developed into sandstone with intercalations of mudstone and weakly developed paleosoil related to the Abu Simbel Formation.
2. Lower Cretaceous rocks are differentiated into Lake Nasser Formation and Sabaya Formation; both mainly developed into coarse grained sandstone with shale and clayey siltstone intercalations.
3. Upper Cretaceous rocks, represented by Kiseiba Formation, developed into fine sandstone with shale, silt, and sandstone intercalation sand bone with phosphate beds.
4. Paleocene rocks, made up of a succession of clastics and reefal limestone intercalations rich in fossils related to the Kurkur Formation.
5. Lower Eocene rocks are differentiated into the Garra Formation and Dungul Formation and are composed mainly of thick limestone beds, partly chalky and occasionally siliceous and dolomitic.
6. Oligocene rocks are developed into dark, low hills.
7. Quaternary rocks; developed into alluvial, lake and playa deposits, as well as Aeolian deposits. The Nubian sandstone rocks in the investigated area represent rocks from Upper Jurassic—Lower Cretaceous to Upper Cretaceous rocks.

### Hydrogeology

**Fig. 4:**
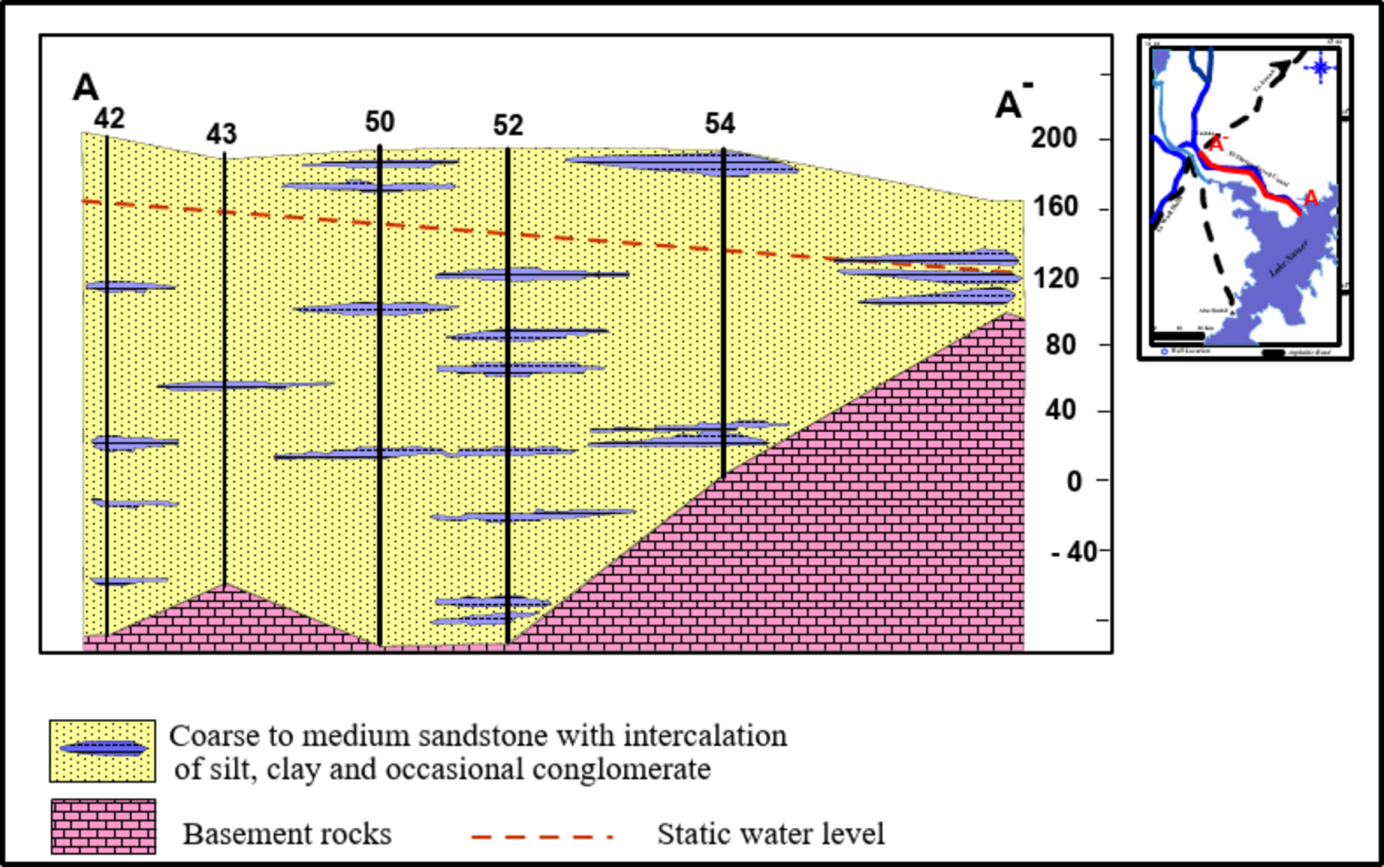
Hydrogeologic cross section for the Nubian Sandstone aquifer after^8^.

The Nubian sandstone constitutes the main rock unit in the subsurface, and they are composed of a course to medium sandstone with intercalations of siltstone clay and occasional conglomerate that rest unconformable on the Precambrian basement rocks at different depths. The sediments of the Nubian Sandstone aquifer were deposited in predominately continental environments. Meandering rivers and deltas were the usual transport mechanisms, which were spatially effective at different times^13^. A hydrogeological cross-section crossing the study area in the SE-NW direction illustrates the composition of the Nubian Sandstone aquifer (Fig. 4).

**Fig. 5:**
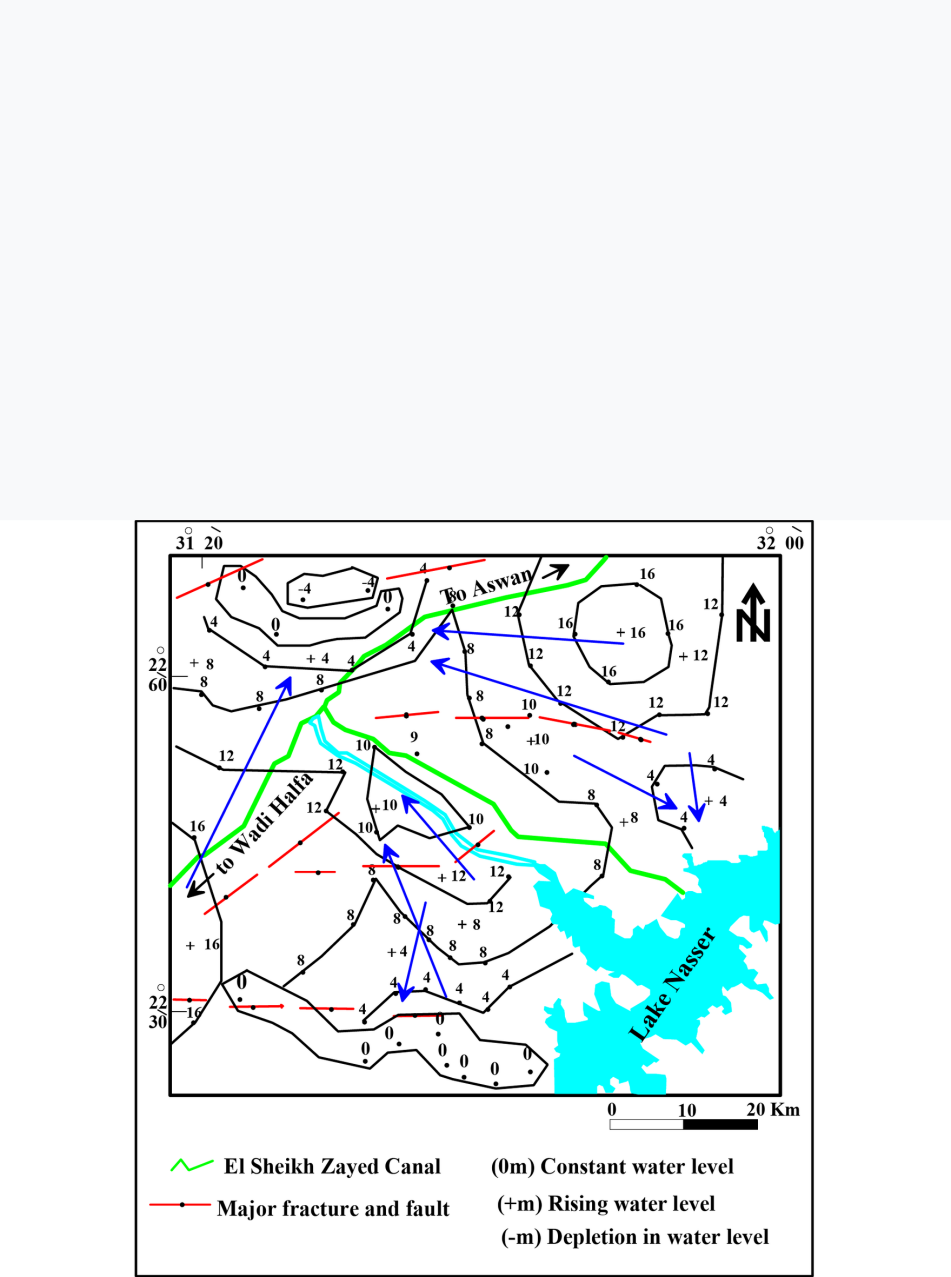
Water table map of the study area after^9^.

The groundwater exists under unconfined and semi-confined conditions. The semi-confined behavior is more detected towards the middle parts of the area, occasionally caused by the occurrence of clay and mudstone layers. The configuration of the basement rocks, which is defined by the geologic structure, governs to a great extent the distribution and thickness of the Nubian series both in vertical and horizontal directions. The thickness of the Nubian sandstone layer varies between 170 and 320 m. Generally, this thickness increases toward the southeastern directions. The depth to basement increases regionally in the southeast and decreases in the northwest and northeast directions, following the same trend of aquifer thickness. The depth of the water table in this aquifer ranges between 20 and 147 m^8^. The groundwater is mainly fossil water, and its age varies between 20,000 and 40,000 years, formed during the long geological history since its formation. This means that the water in storage was recharged in situ during one or more of the rainy periods of late Pleistocene times^14^. The recharge of the Nubian aquifer is from three sources:

(a) Water leakage from Lake Nasser, (b) Recent return flow irrigation water, (c) Infiltration of the rain water during wet periods in the past (Fossil water)^13^. According to the water table map (Fig. 5), the main groundwater flow direction is detected from the direction of Lake Nasser at the southeast towards the middle part of the study aquifer surrounding Tushka depression. According to^8^, the groundwater float charge is 0.0044 m/day on the northeastern part; however, inside the center parts, the groundwater float charge equals 0.0549 m/day. The transmissivty ranges between 336.9 m^2^/day and 2802 m^2^/day with an average value of 1569.45 m^2^/day. The hydraulic conductivity varies from 4.18 to 44.3 m/day with an average value of 24.24 m/day. The increase in hydraulic conductivity values follows the same trend of transmissivity regarding the saturated thickness and clay content. The values of hydraulic conductivity and transmissivity classify the Nubian aquifer as ranging between moderate and high potential aquifer^7^.

## Surface water-groundwater relationship

All hydrologists who studied the problem of seepage from Lake Nasser in the early stage of the project (1963) have recorded seepage from the lake to adjacent Nubian sandstone aquifer based on permeability measurements. After that time estimated the amount of seepage in the Nubian aquifer as equal to 2000 million m^3^/year at a reservoir level of 180 m and 600 million m^3^/year at a level of 150 m, and no seepage at all at a level of 120 m.

^15^proved the presence of hydraulic connections between Lake Nasser and the concerned aquifer at variety distances in four sectors of the study area. Later on^16^, came to the conclusion that the maximum distance from the lake beyond which its contribution becomes negligible is about 50 km.

## Materials and methods

In April 2023, water samples were collected from 25 sites in the study area under natural flow conditions (i.e., during the high flow season). Sample locations were selected from various parts of the study area (Fig. 1) including Lake Nasser stem (sample No. 25) and 24 drilled wells. The location of each sample was determined using a handheld Global Positioning System (GPS) (GARMIN, Germany) (Fig. 1). The temperature, pH, and electrical conductivity (EC) were measured directly on the field using portable meters (JENWAY Model 430). At each location, two sample bottles were collected. Each bottle was cleaned well with the sample water before sampling. One bottle was collected for anions and nitrate analysis. The second bottle was collected and filtered on-site using 0.45 micron filters and acidified with concentrated nitric acid (HNO_3_) for the preservation of dissolved metals and cations. The groundwater analyses were performed in the Hydrogeochemistry Department, Desert Research Center (DRC), Egypt, according to the methods adopted by the^17^. Total Dissolved Solids (TDS) were measured by the evaporation method, Calcium (Ca) and magnesium (Mg) were attempted titrimetrically using standard Na_2_-EDTA, Chloride (Cl) by standard AgNO_3_ titration, Carbonate (CO_3_) and bicarbonate (HCO_3_) by titration with H_2_SO_4_, sodium (Na) and potassium (K) by flame photometry, sulfate (SO_4_), is determined by the turbidity method using UV/Visible spectrophotometer, Unicam model UV4-200 (UK); wavelength 420 nm. The concentrations of the resulting chemical constituents are reported in milligrams per liter (mg/l). Also, a milliequivalent per liter (meq/l) concentration is calculated. The error percentage of the charge balance (CEB) is calculated as the difference between the total cations and the total anions, normalized to the sum of the total cations plus the total anions (expressed as millequivalents/l). The percent charge-balance error is usually expressed as (Eq. 1), according to^18,19^.

1 $$\left( {{\text{CBE}}} \right){\text{ = }}\left( {\frac{{\sum \: {\text{Cations}}\,{\text{ - }}\sum {{\text{Anions}}} }}{{\sum \: {\text{Cations}}\,{\text{ + }}\sum \: {\text{Anions}}}}} \right) \times {\text{100}}$$

If the CBE is less than 5%, the analysis is considered acceptable. The charge balance errors observed in the studied samples range from − 4.2 to + 3.95, with an average of -0.23 (Table 1). Concentrations of heavy metals (Ag, Al, B, Ba, Cd, Co, Cr, Cu, Fe, Mn, Mo, Ni, Pb, Si, Sr, V and Zn) in all collected samples were done, (Table 2) using Inductively Coupled Argon Plasma, ICAP 6500 Duo, Thermo Scientific, England General solutions of a thousand mg/L, Merck, Germany, have been used as inventory solutions for tool standardization. Nitrate concentration in all the collected samples is determined colorimetrically by the chromotropic acid (CTA) method using UV/visible spectrophotometer, Unicam model UV4-200 at 430 nm. The results were tabulated in mg/l (Table 2) for interpretation.

**Table 1:** Hydrochemical analyses data of the investigated water samples in mg/l.

Sample no.	pH	E.C µs/cm	TDS mg/l	Ca^2+^	Mg^2+^	Na^+^	K^+^	CO_3_^2−^	HCO_3_^−^	SO_4_^2−^	Cl^−^	e%
1	7.4	914	601.73	96.99	26.70	53.33	2.00	0.00	167.75	192.81	118.26	− 3.55
2	7.6	1726	1097.16	175.00	20.27	150.00	2.00	0.00	122.00	360.00	313.65	− 3.86
3	7.6	822	492.31	26.26	9.11	130.00	2.00	0.00	61.00	120.29	160.85	− 1.74
4	6.5	659	394.54	41.60	4.11	86.50	1.00	0.00	33.55	21.73	200.53	− 3.54
5	6.9	768	452.66	81.32	11.29	50.00	2.00	0.00	111.15	151.25	87.98	− 1.63
6	7.5	481	349.87	54.27	9.21	42.00	2.00	0.00	76.25	16.34	146.53	− 3.42
7	6.9	800	453.78	45.79	10.46	108.50	1.00	0.00	42.70	25.45	221.10	2.77
8	6.6	671	383.31	75.41	5.22	60.00	1.00	0.00	30.50	2.04	210.82	2.56
9	6.4	541	348.10	26.50	8.15	75.00	1.00	0.00	45.75	36.12	139.40	− 1.42
10	6.5	543	325.82	7.18	30.00	65.00	2.00	0.00	61.00	10.40	154.26	2.60
11	7	466	266.70	15.64	9.01	64.00	2.00	0.00	60.47	55.00	67.00	3.95
12	5.8	513	326.39	25.42	5.81	78.04	2.00	0.00	48.80	18.32	149.11	− 1.83
13	5.8	582	341.61	31.91	8.61	82.00	2.00	0.00	45.75	37.90	139.96	3.79
14	6.3	555	308.11	18.36	6.23	76.17	2.00	0.00	54.90	24.08	128.55	− 2.37
15	6.7	492	295.42	14.36	3.91	79.00	2.00	0.00	79.30	15.38	113.12	− 3.04
16	6.6	591	373.41	32.34	7.57	98.65	2.00	0.00	54.90	24.44	169.68	3.02
17	6.7	532	346.44	18.93	7.75	87.09	2.00	0.00	73.20	54.54	123.40	− 3.50
18	6.7	538	358.03	30.08	7.93	94.00	1.00	0.00	61.00	29.16	149.11	3.77
19	6.8	564	411.56	3.90	8.09	139.73	2.00	0.00	54.90	40.48	169.68	3.42
20	6.3	679	502.18	52.10	9.32	122.00	1.00	0.00	51.85	55.18	221.10	2.75
21	6.4	1035	679.52	126.70	11.51	108.00	2.00	0.00	39.65	70.40	329.08	2.66
22	6.7	725	415.46	42.58	8.91	92.50	2.00	0.00	45.75	9.85	226.24	− 2.82
23	6.8	431	271.75	15.02	3.94	68.00	2.00	0.00	79.30	30.34	88.98	− 4.20
24	6.3	815	523.57	49.96	12.34	124.32	2.00	0.00	176.90	128.92	102.84	2.77
25*	7.2	242.2	154.33	32.82	8.78	8.00	1.00	0.00	106.75	20.51	25.71	− 2.99

25* Lake Nasser water sample.

**Table 2:** Concentrations of minor and trace constituents in mg/l in the study area.

Sample no.	Ag	Al	B	Ba	Cd	Co	Cr	Cu	Fe	Mn	Mo	Ni	Pb	Si	Sr	V	Zn	NO_3_
1	N.D	0.33	N.D	N.D	N.D	0.0001	0.017	0.001	0.04	0.0016	N.D	0.78	0.007	4.43	0.44	N.D	0.01	21.7
2	N.D	0.12	N.D	0.037	N.D	N.D	0.02	N.D	1.21	0.016	N.D	0.16	0.0027	4.300	0.950	N.D	0.02	8.4
3	N.D	0.07	N.D	0.04	N.D	N.D	0.01	N.D	6.3	0.08	N.D	0.04	N.D	0.670	0.310	N.D	0.18	5.6
4	N.D	0.28	N.D	0.17	N.D	0.0004	0.024	N.D	10.38	0.29	N.D	0.17	0.0016	0.610	0.290	N.D	0.28	9.8
5	N.D	N.D	N.D	0.05	N.D	N.D	0.15	0.02	0.25	0.002	N.D	0.014	0.003	4	0.35	N.D	0.008	8.4
6	N.D	0.317	N.D	0.049	N.D	0.0006	0.289	0.478	25.92	0.4566	N.D	0.0105	0.0094	1.110	0.456	N.D	0.405	11.9
7	N.D	0.329	N.D	0.066	N.D	0.005	0.016	0.014	18.31	0.449	N.D	0.016	0.0047	0.456	0.3455	N.D	0.12	N.D
8	N.D	0.12	N.D	0.06	N.D	0.0001	0.0054	0.033	8.55	0.55	N.D	0.0057	0.27	N.D	0.33	N.D	0.15	3.5
9	N.D	0.17	N.D	0.046	N.D	0.0005	0.008	0.012	8.63	0.613	N.D	0.007	0.007	N.D	0.128	N.D	0.034	29.4
10	N.D	0.12	N.D	0.034	N.D	N.D	0.0017	0.0043	4.66	0.298	N.D	0.003	0.004	N.D	0.265	N.D	0.094	21
11	N.D	0.453	N.D	0.027	N.D	N.D	0.0108	0.006	10.64	0.228	N.D	0.007	0.005	1.11	0.07	N.D	0.06	11.2
12	N.D	0.28	N.D	0.055	N.D	0.0002	0.006	0.0026	8.25	0.17	N.D	0.005	0.0018	0.21	0.238	N.D	0.065	14
13	N.D	0.207	N.D	0.05	N.D	0.0006	0.0144	0.008	11.07	0.427	N.D	0.009	0.002	0.046	0.219	N.D	0.098	4.2
14	N.D	0.36	N.D	0.024	N.D	N.D	0.003	0.015	14.97	0.39	N.D	0.006	0.01	0.23	0.075	N.D	0.087	9.1
15	N.D	0.27	N.D	0.05	N.D	0.0006	0.0016	0.0114	13.68	0.19	N.D	0.004	0	0.326	0.11	N.D	0.054	13.3
16	N.D	0.21	N.D	0.05	N.D	0.0001	0.0001	0.008	8.36	0.24	N.D	0.004	0.002	N.D	0.219	N.D	0.088	2.1
17	N.D	0.27	N.D	0.023	N.D	0.0003	0.005	0.004	13.14	0.26	N.D	0.005	0.005	0.17	0.09	N.D	0.05	2.1
18	N.D	0.149	N.D	0.02	N.D	0.0003	N.D	N.D	8.57	0.33	N.D	0.0037	0.005	N.D	0.14	N.D	0.025	7
19	N.D	0.15	N.D	0.05	N.D	0.006	0.007	0.009	15.14	0.39	N.D	0.006	0.02	N.D	0.2	N.D	0.048	4.2
20	N.D	0.125	N.D	0.046	N.D	0.0001	0.0026	0.002	7.86	0.745	N.D	0.002	0.005	N.D	0.28	N.D	0.19	6.3
21	N.D	0.59	N.D	0.093	N.D	0.0009	0.009	0.012	8.9	0.911	N.D	0.004	0.008	0.43	0.62	N.D	0.426	N.D
22	N.D	0.318	N.D	0.05	N.D	0.0006	0.008	0.01	9.24	0.29	N.D	0.0041	0.01	N.D	0.37	N.D	0.2	N.D
23	N.D	0.5	N.D	0.1	N.D	0.0004	0.0023	N.D	9.05	0.17	N.D	0.0027	0.003	0.51	0.1	N.D	0.08	13.3
24	N.D	0.2	N.D	0.04	N.D	N.D	0.015	N.D	0.32	0.014	N.D	0.002	0.001	3.99	0.35	N.D	0.007	9.8
25*	N.D	0.21	N.D	0.02	N.D	0.0002	N.D	N.D	0.27	0.01	N.D	0.0007	N.D	2.05	0.169	N.D	0.0018	1.4

25* Lake Nasser water sample.

The groundwater samples’ chemical data was interpreted using a variety of techniques, including graphs, ion correlations, statistical analysis, software, and geochemical modeling. A Piper plot^20^ was used to identify the various chemical types of the studied groundwater samples. Hydrochemical coefficients (ion ratios) are correlations between the components of the dissolved groundwater that show the stoichiometric balance of the different ions and suggest the plausible hydrochemical processes affecting the solution. These ratios are useful for determining the chemical changes that significantly affect the quality of the groundwater, such as leaching and dissolution, contamination, mixing, and ion exchange. The ion coefficients that are of certain importance in this respect are Na/Cl, Ca/Mg, and SO_4_/Cl. The hydrochemical program PHREEQC^21^ is used to perform the mass balance simulation, which shows chemical reactions and changes in chemical nature, such as the dissolution/precipitation of minerals and gases in the groundwater flow channel. The groundwater system’s ability for minerals to dissolve or precipitate is demonstrated by the results of Saturation Index (SI) values^21^. The following equation would be used to calculate the saturation indices for the minerals that should exit or enter the solution: SI is equal to log (IAP/Kt), where Kt is the equilibrium solubility constant of the mineral and IAP is the ion-activity product in a diffused charged species solution. A negative value on the SI for any mineral phase means that the water is under saturated regarding that mineral and will disintegrate until equilibrium is attained, while a positive sign means that the water is oversaturated concerning that mineral and the mineral will depart the solution or be precipitated. SPSS, or Statistical Package, is a statistical program that can be used to analyze any type of scientific data to determine the pattern of variation among variables or compress data into a manageable number of components or variables. The hydrochemical parameters (such as TDS, Ca, Mg, Na, HCO_3_, SO_4_, and Cl^−^) are among the variables used to identify the hydrogeochemical processes and the mineralization of the groundwater.

In the hypothetical salts identification, the ions of the strong acids (Cl^−^ and SO_4_^2−^) form chemical combinations with alkalis (Na^+^ and K^+^), and the rest of the acid radicals combine with the alkaline earths (Ca^2+^ and Mg^2+^). If the cations of alkalis and alkaline earths are surplus in water, they well combine with the weak acids (CO_3_^2−^ and HCO_3_^−^). To clarify such combinations, the relations between cations and anions in the investigated waters are illustrated in the form of bar graphs as outlined by^22^ and^23^. In this method of representation, the concentrations of dissolved cations and anions, expressed in me/l, are represented by a vertical bar graph, the height of which is equivalent to the total concentration of either cations or anions. Each bar is subdivided into two longitudinal halves; the cations are plotted on the left half while the anions are plotted on the right one. Vertically, each bar is subdivided into several sections representing the present concentrations of the major cations and anions. The deduced salt combinations on qualitative and quantitative bases are appended and discussed. The role of the environmental isotopes in groundwater hydrogeochemistry is very important in determining the origin of groundwater and effective processes affecting groundwater quality. During the present study, an approach was made to focus on the isotopic characteristics of water samples. To achieve this goal, 9 representative groundwater samples from the Nubian Sandstone aquifer (NSSA), and the lake Nasser were taken from^9^, and the pure Nubian sandstone sample was taken from^24^.

A hypothetical blending model is proposed to demonstrate the contribution percent of the 2 sources acquired through every pattern relying on the topographic and the geological state of affairs of every sample according to the following equation:

$$\updelta {\text{ 18O}}={\text{aX}}+{\text{b }}\left( {{\text{1}} - {\text{X}}} \right)$$

Where (a) and (b) are the parameters illustrating δ 18O‰ of the main feeding sources (in our case: paleowater and Lake Nasser water, respectively).

δ 18O: is the value of Oxygen-18 content X: is the contribution percent of paleowater 1-X; is the contribution percent of Lake Nasser water.

## Results and discussions

### Groundwater salinity

**Fig. 6:**
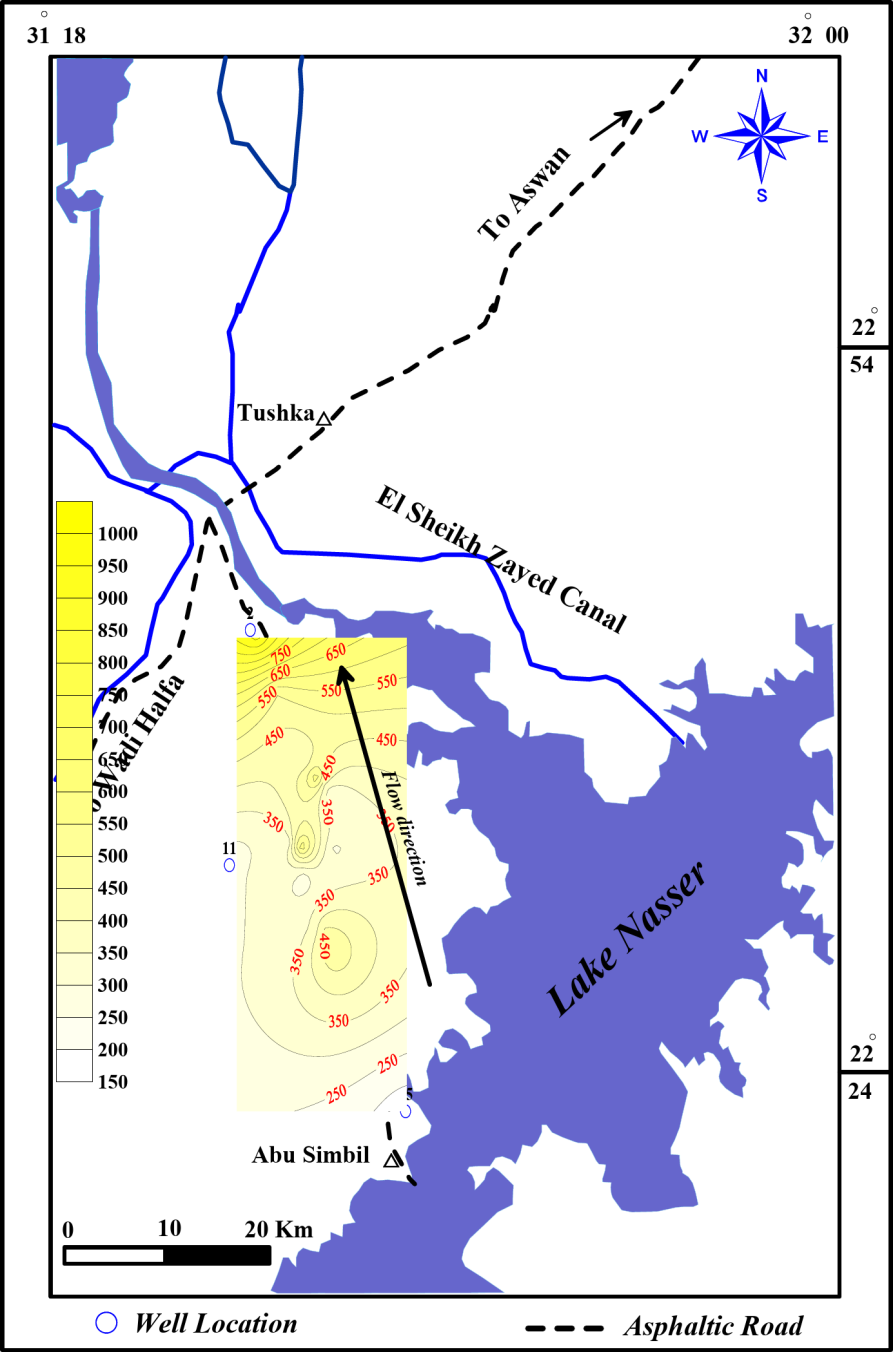
Iso-salinity contour map of the groundwater in the study area.

According to^25^, the groundwater in the study area is classified into two main categories of total salinity: fresh water where the TDS values are less than 1000 mg/l which represented the majority of the groundwater samples (96%), while only one sample (4%) considers a brackish groundwater sample according to^25^, where the TDS value is 1097 mg/l (Table 1). The iso-salinity contour map indicates that the salinity decreases towards Lake Nasser and southeastern parts of the study area (Fig. 6). It confirms the main direction of groundwater flow from the Lake Nasser towards the northwestern part of the study area.

### Physicochemical parameters

The pH values of the selected groundwater samples vary from 5.8 in wells No. 12 and 13 in the center to 7.6 in well No. 3 on the northern side, with a median value of 7.3. The pH values change from slightly acidic in the central parts to neutral in the northern side.

### Groundwater mineralization processes

**Fig. 7:**
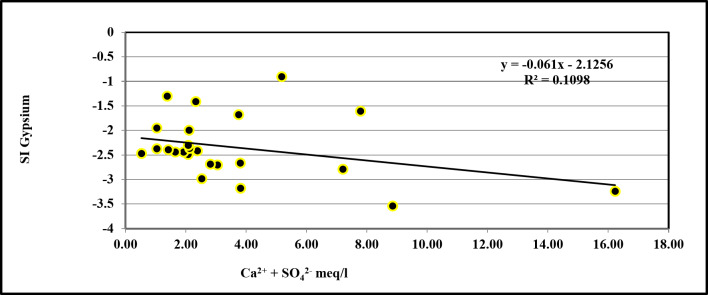
Plot of SI of Gypsum versus of (Ca^2+^ + SO_4_^2−^meq/l) in the Groundwater samples.

^31^ suggested two Chloro-alkaline indices CAI-1 and CAI-2, for the interpretation of ion exchange between groundwater and the environment:

Pearson Correlation analysis was performed using the SPSS software^26^ to identify the correlation between the major elements versus total dissolved solids that contribute to groundwater mineralization (Table 3). The resulting correlations indicate that the referenced elements contribute significantly to groundwater salinization in this aquifer. These correlations in the case of the groundwater wells show a strong correlation between the values of Ca^2+^, Cl^−^, and SO_4_^2−^ with the TDS values (0.869 for Ca^2+^, 0.714 for Cl^−^ and 0.857 for SO_4_^2−^) and a moderate correlation between Na^+^ values with TDS values (0.623); there is also no correlation designated in the case of HCO_3_ values with TDS (0.334). We created correlations between Ca^2+^ & SO_4_^2−^, Mg^2+^ & SO_4_^2−^, and Ca^2+^ & Mg^2+^ to show the many mechanisms causing this mineralization (Table 3). In the groundwater wells, the strong positive correlation between Ca^2+^ and SO_4_^2−^ (0.754) shows that the dissolution of gypsum is the major process of groundwater salinization^27^. This dissolution is also confirmed with the majority of samples by the low saturation indices concerning these minerals (from − 3.54 to − 0.903, Table 4) and shows a positive relationship (R^2^ = 0.1098) when correlated with the concentrations of Ca^2+^ + SO_4_^2−^meq/l (Fig. 7). As a result, we may deduce another source of SO_4_^2−^, which is most likely caused by the dissolution of the epsomite mineral^28^. This is also confirmed by the positive correlation between Mg^2+^ and SO_4_^2−^ in the groundwater (0.513) (Table 3).

**Table 3:** Correlation coefficient of chemical ions of the groundwater in the study area.

	TDS	Na^+^	Mg^2+^	Ca^2+^	Cl^−^	SO_4_^2−^	HCO_3_^−^
TDS	1						
Na^+^	0.623	1					
Mg^2+^	0.459	0.015	1				
Ca^2+^	0.869	0.220	0.386	1			
Cl^−^	0.714	0.626	0.141	0.616	1		
SO_4_^2−^	0.857	0.383	0.513	0.754	0.267	1	
HCO_3_^−^	0.334	− 0.070	0.480	0.344	− 0.344	0.644	1

**Table 4:** Saturation indices of the groundwater minerals in the investigated area.

Sample no.	Calcite	Aragonite	Dolomite	Siderite	Gypsum	Anhydrite	Gibbs	Hematite	Goethite	Manganite
1	0.111	− 0.029	0.054	− 1.068	− 1.301	− 1.499	1.967	10.063	3.673	− 11.007
2	0.358	0.218	0.175	0.419	− 0.903	− 1.097	1.279	14.236	5.696	− 9.457
3	− 0.597	− 0.734	− 1.228	1.01	− 1.951	− 2.129	0.913	16.209	6.472	− 8.653
4	− 1.715	− 1.852	− 3.993	− 0.067	− 2.44	− 2.615	2.481	10.276	3.457	− 11.305
5	− 0.557	− 0.695	− 1.553	− 0.842	− 1.411	− 1.594	–	9.104	2.975	− 12.348
6	− 0.317	− 0.458	− 1.008	1.586	− 2.469	− 2.671	1.853	16.353	6.861	− 8.177
7	− 1.175	− 1.311	− 2.541	0.67	− 2.373	− 2.541	2.148	13.188	4.837	− 9.971
8	− 1.332	− 1.467	− 3.353	− 0.034	− 3.24	− 3.391	1.861	11.106	3.606	− 10.751
9	− 1.881	− 2.02	− 3.857	− 0.121	− 2.393	− 2.581	2.407	9.204	3.095	− 11.287
10	− 1.892	− 2.03	− 2.728	0.139	− 3.54	− 3.72	2.179	9.437	3.114	− 11.298
11	− 1.398	− 1.537	− 2.621	0.672	− 2.416	− 2.604	2.35	12.918	4.945	− 9.959
12	− 2.487	− 2.627	− 5.214	− 0.728	− 2.688	− 2.887	2.549	5.338	1.311	− 13.632
13	− 2.43	− 2.571	− 5.03	− 0.642	− 2.305	− 2.503	2.386	5.524	1.397	− 13.273
14	− 2.084	− 2.224	− 4.234	0.072	− 2.705	− 2.902	2.821	8.867	3.054	− 11.784
15	− 1.662	− 1.805	− 3.521	0.55	− 2.984	− 3.197	2.642	10.767	4.255	− 10.897
16	− 1.599	− 1.742	− 3.464	0.057	− 2.489	− 2.704	2.592	9.656	3.722	− 11.12
17	− 1.613	− 1.756	− 3.252	0.467	− 2.366	− 2.58	2.653	10.677	4.225	− 10.776
18	− 1.482	− 1.625	− 3.182	0.213	− 2.439	− 2.654	2.384	10.24	4.028	− 10.688
19	− 2.325	− 2.467	− 3.969	0.508	− 3.179	− 3.393	2.298	11.339	4.563	− 10.327
20	− 1.741	− 1.884	− 3.861	− 0.314	− 1.995	− 2.207	2.45	7.819	2.767	− 11.55
21	− 1.455	− 1.597	− 3.581	− 0.352	− 1.606	− 1.818	3.101	8.483	3.099	− 11.187
22	− 1.46	− 1.602	− 3.218	0.125	− 2.79	− 2.998	2.67	10.568	4.076	− 10.712
23	− 1.551	− 1.693	− 3.314	0.464	− 2.665	− 2.877	2.835	11.051	4.383	− 10.637
24	− 1.237	− 1.379	− 2.705	− 1.221	− 1.678	− 1.885	2.623	5.053	1.297	− 13.337

Ion-exchange index **[rCl**^**−**^**– r(Na**^**+**^**+K**^**+**^**)]/rCl**^**−**^ has either negative or positive values (Table 5). The negative values mean that alkaline earth (Ca^2+^ and Mg^2+^) in water replaces alkalis (Na^+^+K^+^) on the surface of clay minerals in the aquifer, and vice versa in the case of the positive values^23,29,30^. 71% of the groundwater samples have positive values of cation exchange index and vice versa in the case of the rest of the samples (29%), which have negative values of cation exchange index.

**Table 5:** Ion ratios of the groundwater samples in the investigated area.

S. no.	rNa^+^/rCl^−^	rCa^2+^/rMg^2+^	rSO_4_^2−^/rCl^−^	rNa/*r*(Na + Cl)	[rCl^−^– *r*(Na^+^+K^+^)]/rCl^−^
1	0.70	2.20	1.20	0.41	0.29
2	0.74	5.24	0.85	0.42	0.26
3	1.25	1.75	0.55	0.55	− 0.26
4	0.67	6.14	0.08	0.40	0.33
5	0.88	4.37	1.27	0.47	0.10
6	0.44	3.58	0.08	0.31	0.55
7	0.76	2.66	0.08	0.43	0.24
8	0.44	8.77	0.01	0.31	0.56
9	0.83	1.97	0.19	0.45	0.16
10	0.88	0.15	0.05	0.47	0.10
11	1.47	1.05	0.61	0.60	− 0.50
12	0.81	2.65	0.09	0.45	0.18
13	0.90	2.25	0.20	0.47	0.08
14	0.91	1.79	0.14	0.48	0.07
15	1.08	2.23	0.10	0.52	− 0.09
16	0.90	2.59	0.11	0.47	0.09
17	1.09	1.48	0.33	0.52	− 0.10
18	0.97	2.30	0.14	0.49	0.02
19	1.27	0.29	0.18	0.56	− 0.28
20	0.85	3.39	0.18	0.46	0.14
21	0.51	6.68	0.16	0.34	0.49
22	0.63	2.90	0.03	0.39	0.36
23	1.18	2.31	0.25	0.54	− 0.20
24	1.86	2.46	0.93	0.65	-0.88

$${\text{CAI-}}1= \left( {{\text{C}}{{\text{l}}^-}- \left( {{\text{N}}{{\text{a}}^+}+ {{\text{K}}^+}} \right)} \right)/{\text{C}}{{\text{l}}^-}$$

$${\text{CAI-}}2 = \left( {\text{Cl}}^{-}-\left( {\text{Na}}^{+}+ {\text{K}}^{+} \right) \right)/\left( {{\text{SO}}_{4}}^{2-}+{{\text{HCO}}_{3}}^{-}+{{\text{CO}}_{3}}^{2-}+{{\text{NO}}_{3}}^{-}\right)$$

**Fig. 8:**
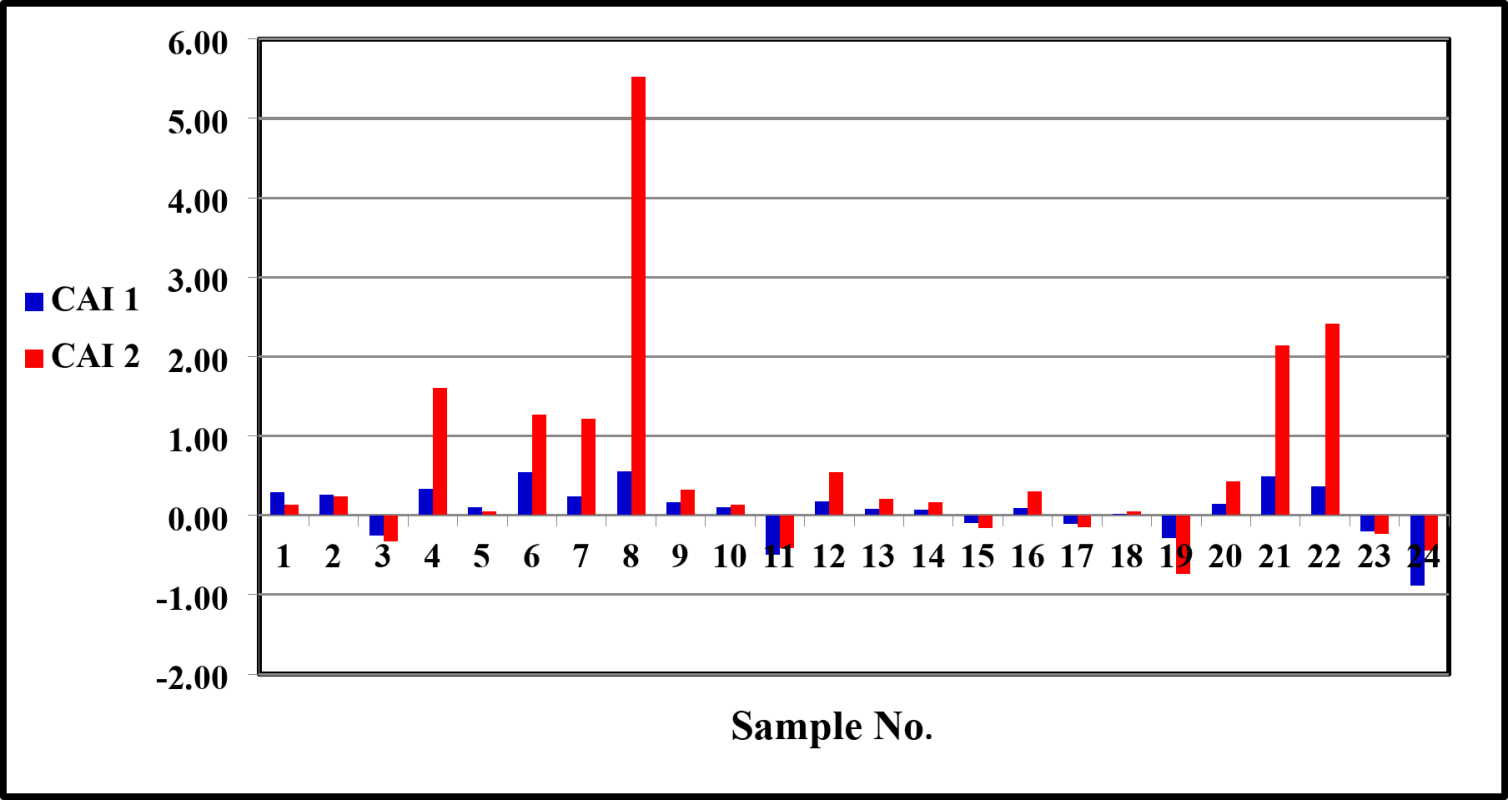
The Chloro - alkaline indices CAI 1 and CAI 2.

All values are expressed in meq/l. Positive Chloro-alkaline indices indicate exchange of Na^+^ and K^+^ from the water with Ca^2+^ and Mg^2+^ from the rocks, while the negative indices inform that there is an exchange of Ca^2+^ and Mg^2+^ from the water with Na^+^ and K^+^ from the rocks^31,32^. The majority of the groundwater samples have positive values of chloro-alkaline indices indicating direct base exchange, depletion of Na^+^, and enrichment of Ca^2+^ and Mg^2+^ (Table 6; Fig. 8).

**Table 6:** Rest of ion ratios of the groundwater samples in the investigated area.

S. no.	rCl/*r*(CO_3_ + HCO_3_)	Ca/Na mmol	HCO_3_/Na mmol	CAI 1	CAI 2	Na/Cl mmol	Ca/Mg mmol
1	1.21	1.04	1.19	0.29	0.14	1.07	1.34
2	4.42	0.67	0.31	0.26	0.24	1.14	3.18
3	4.54	0.12	0.18	− 0.26	− 0.33	1.92	1.06
4	10.28	0.28	0.15	0.33	1.61	1.03	3.72
5	1.36	0.93	0.84	0.10	0.05	1.35	2.65
6	3.31	0.74	0.68	0.55	1.26	0.68	2.17
7	8.91	0.24	0.15	0.24	1.21	1.17	1.61
8	11.89	0.72	0.19	0.56	5.53	0.68	5.32
9	5.24	0.20	0.23	0.16	0.33	1.28	1.20
10	1.58	0.06	0.35	0.10	0.13	1.36	0.09
11	1.91	0.14	0.36	− 0.50	− 0.41	2.27	0.64
12	5.26	0.19	0.24	0.18	0.54	1.24	1.61
13	5.26	0.22	0.21	0.08	0.20	1.39	1.36
14	4.03	0.14	0.27	0.07	0.17	1.41	1.08
15	2.45	0.10	0.38	− 0.09	− 0.16	1.66	1.35
16	5.32	0.19	0.21	0.09	0.31	1.38	1.57
17	2.90	0.12	0.32	− 0.10	− 0.15	1.68	0.90
18	4.21	0.18	0.24	0.02	0.05	1.50	1.40
19	5.32	0.02	0.15	− 0.28	− 0.74	1.96	0.18
20	7.34	0.24	0.16	0.14	0.43	1.31	2.06
21	14.28	0.67	0.14	0.49	2.14	0.78	4.05
22	8.51	0.26	0.19	0.36	2.41	0.97	1.76
23	1.93	0.13	0.44	− 0.20	− 0.23	1.82	1.40
24	1.00	0.23	0.54	− 0.88	− 0.45	2.88	1.49

### Saturation indices (SI)

Saturation Indices (SI) of the resulting minerals were determined to understand the interaction of rocks with flowing groundwater^21^. The PHREEQC hydrochemical program is used to perform the mass balance simulation, which shows changes in chemical nature and chemical reactions, such as the dissolution/precipitation of minerals in the groundwater flow channel. The chemical data (Tables 1 and 2) is used to calculate these minerals. Table 4 shows the SI calculated values of the selected minerals. The negative value of SI for any mineral means that the water is under saturated while a positive sign means that the water is oversaturated concerning that mineral and the mineral will leave the solution or be precipitated. Table 4 illustrates gypsum, anhydrite and manganite dissolution with negative SI values, whereas iron minerals (hematite and goethite) are supersaturated with positive values. Carbonate minerals (calcite, dolomite, and aragonite) are completely insoluble in groundwater, and therefore their values fluctuate positively and negatively around zero. This confirms the role of the various geochemical processes, which were previously identified by studying the relationships of ions with each other in the enrichment or depletion of groundwater with the elements related to these minerals.

### Hypothetical salts assemblages

According to^22^ and^33^, the combination of the major anions and cations reveals the formation of six groups (I, II, III, IV, V, and VI) of hypothetical salts in the groundwater samples (Table 7). The majority of the groundwater samples (64%) are characterized by the assemblages I, II, III, IV and V (less and intermediate stages). Where, 12% of the Nubian Sandstone groundwater samples are characterized by hypothetical salt assemblage II that is similar to the Nubian water. Furthermore 16% of the groundwater samples are characterized by the assemblage I that is similar to the Lake Nasser water this confirms that there is a recharge from Lake Nasser^9^. Assemblages III, IV, and V reflecting some contribution of cation exchange process as well as leaching and dissolution of terrestrial salts (continental facies groundwater, two or three sulfates salts and/or two carbonate salts) by local infiltration of rain water during Pluvial and post Pluvial times^9^. The rest of samples (36%) are characterized by the assemblage (VI) (more advanced stage, three chloride salts). In conclusion, the hypothetical salt assemblages indicate meteoric water origin influenced by leaching and dissolution of terrestrial salts. From all previous discussions, it can be concluded that there is a recharge from Lake Nasser to the Nubian aquifer.

**Table 7:** Combinations of the hypothetical salts in the groundwater.

Assemblages of hypothetical salts in the study area	% of water samples	Sample no.
I-NaCl, Na_2_SO_4_, NaHCO_3_, Mg(HCO_3_)_2_ and Ca(HCO_3_)_2_	16	15, 19, 23, and 25
II-NaCl, Na_2_SO_4_, MgSO_4_, Mg(HCO_3_)_2_ and Ca(HCO_3_)_2_	12	11, 17, and 24
III-NaCl, Na_2_SO_4_, MgSO_4_, CaSO_4_ and Ca(HCO_3_)_2_	4	3
IV-NaCl, MgCl_2_, MgSO_4_, Mg(HCO_3_)_2_ and Ca(HCO_3_)_2_	4	10
V-NaCl, MgCl_2_, MgSO_4_, CaSO_4_ and Ca(HCO_3_)_2_	28	1, 2, 5, 9, 13, 14 and 18
VI-NaCl, MgCl_2_, CaCl_2_, CaSO_4_ and Ca(HCO_3_)_2_	36	4, 6, 7, 8, 12, 16,20, 21 and 22

### Hydrochemical facies and evolution

**Fig. 9:**
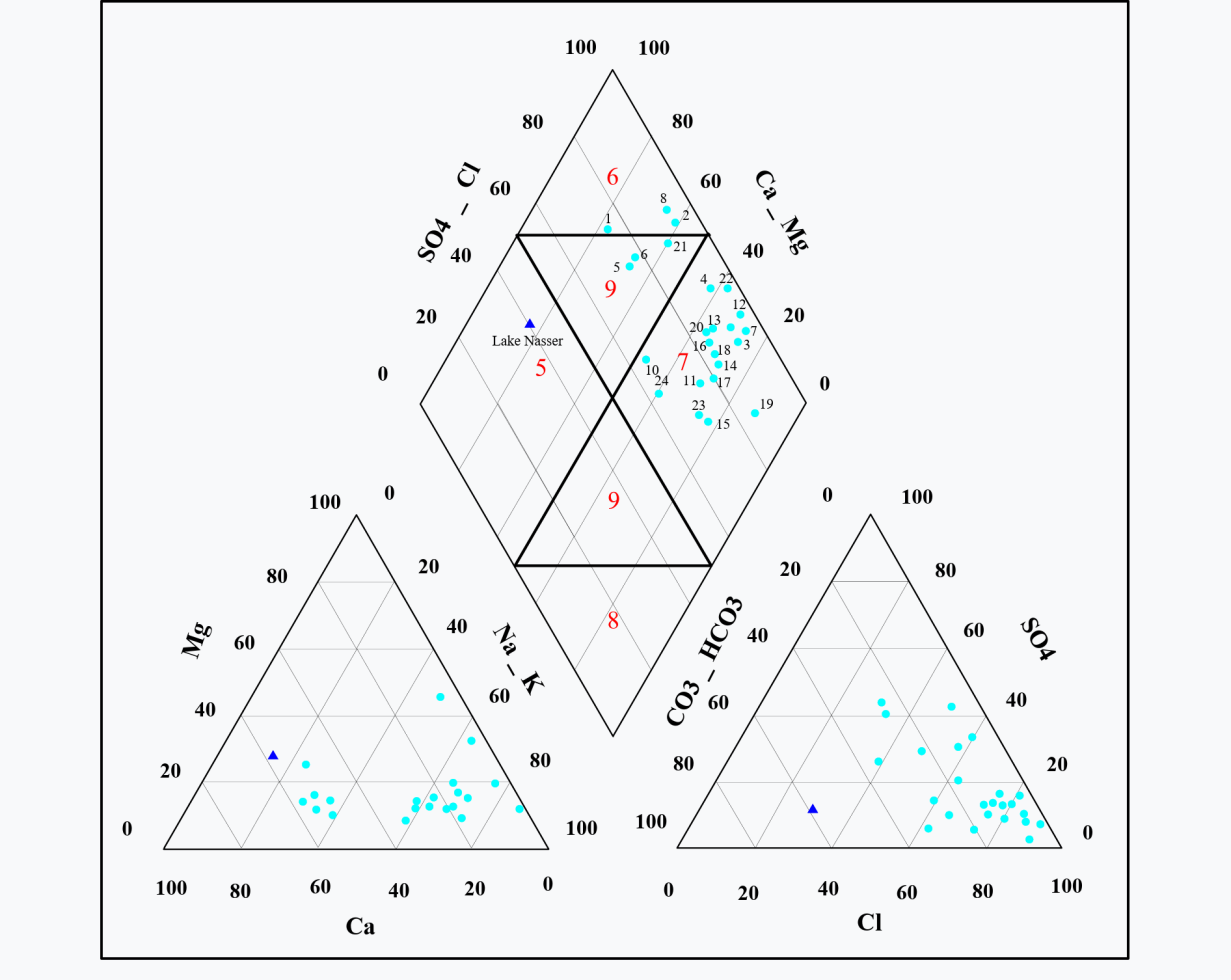
Piper trilinear diagram for groundwater and surface samples in the study area.

The results of the analyzed water samples were plotted on Piper’s tri-linear diagram^20^ as presented in Fig. 9, which shows that three groundwater types can be identified: Na-Cl type, Ca-Cl type, and mixed Ca-Mg-Cl type, which are predominated in the study area. The majority (75%) of the groundwater samples are located in sub-area 7 {Noncarbonate alkali (Na^+^+K^+^) Cl and (Na^+^+K^+^)_2_ SO_4_}, where the groundwater is dominated by alkalis and strong acids (Na-Cl type, primary salinity). This results from the leaching and dissolution of terrestrial salts. Few of the groundwater samples (13%, samples No. 5, 6, and 21) are located in sub-area 9 where no one cation - anion pair exceeds 50%. This type of groundwater has mixed facies, which exhibits in a few groundwater samples affected by Lake Nasser water, paleowater, and terrestrial facies (Ca-Mg-Cl type). The Lake Nasser sample is located in sub-area 5 (carbonate hardness, temporary hardness, or secondary alkalinity) exceeds 50%; that is, chemical properties of the surface water are dominated by alkaline earth and weak acids {CaMg (HCO_3_)_2_}. The rest of the groundwater samples (12%, samples No. 1, 2, and 8) are located in sub-area 6 (noncarbonate hardness or secondary salinity), exceeding 50%, Ca-Cl type, indicates the contribution of paleowater^9^.

### Ion ratios

#### (rNa+/rCl−)

**Fig. 10:**
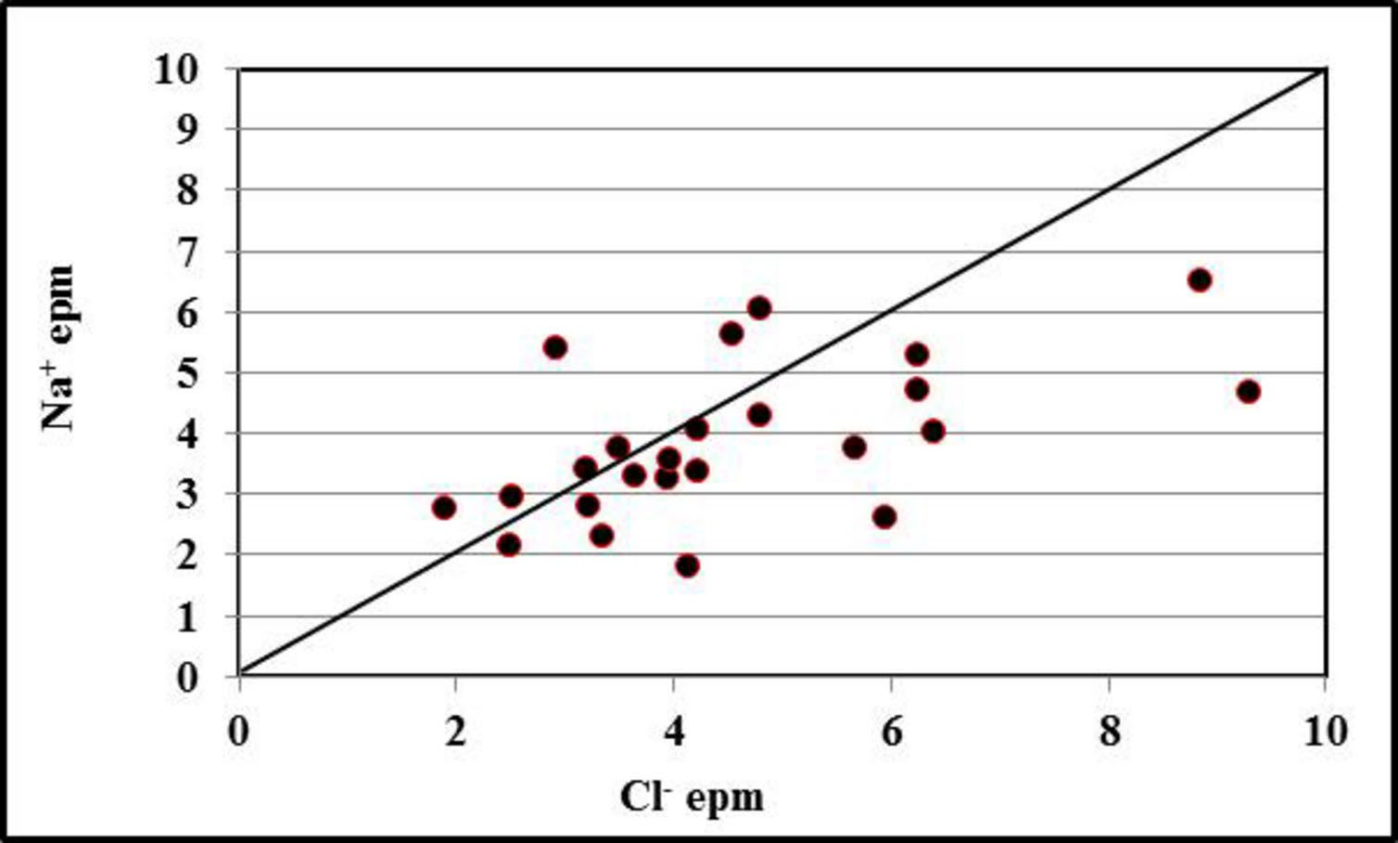
Relationship between the concentrations of Na^+^ vs. C1^−^.

The values of the hydrochemical coefficient (rNa/rCl) for the majority of the groundwater samples (71%) are less than one (Table 5). This reflects the effects of continental facies. The rest of the groundwater samples (29%) have values of (rNa/rCl) more than one; this reflects that sodium is predominately over chloride^34,35^. used the Na^+^ vs. C1^−^ diagram to give an explanation for the contribution of Na^+^ and C1^−^ to the groundwater via the dissolution of evaporate deposits. According to Fig. 10, the groundwater sampling points fall alongside the equiline of Na^+^:C1^−^ (1:1) if the evaporate dissolution occurs. In the present study, the groundwater sampling points (71%) and (29%) are scattered towards the C1^−^ and Na^+^ ions, respectively, which indicate the alternative sources of these ions^33^. While 29% of the samples plotted above the equiline, thus reveal that sodium is reduced from groundwater due to the cation exchange process (Fig. 10), and 71% of the saples plotted below the equiline, thus suggesting the absence of halite dissolution^36^.

**Fig. 11:**
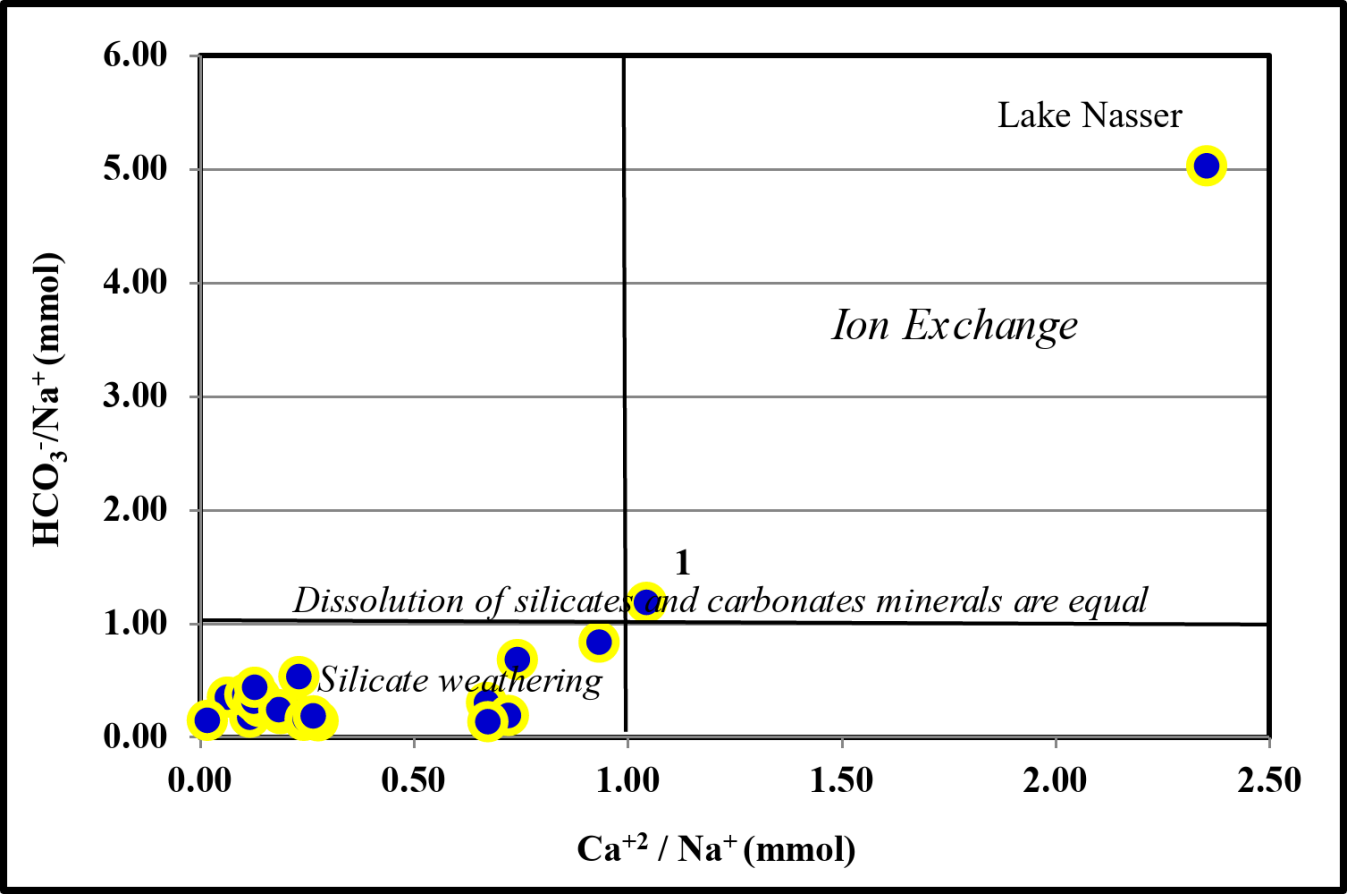
Plot of (HCO_3_^−^/Na^+^) vs. (Ca^2+^/Na^+^) mmol/l.

The other sources of Na^+^ and K^+^ ions can be interpreted as cation exchange and/or silicate weathering sources from the hydrochemical ratios. The silicate weathering as a source of (Na^+^& K^+^) can be declared by the relations of Ca^2+^/Na^+^ versus HCO_3_^−^/Na^+^ molar ratio (Fig. 11).

All the groundwater samples show decreases of Ca^2+^/Na^+^ and HCO_3_^−^/Na^+^. This could be explained as silicate weathering, except sample No. 1 and the lake Nasser sample (Sample No. 25) show ion exchange^37^.

#### (rCa2+/rMg2+)

Values of the hydrochemical coefficient rCa/rMg in the groundwater samples range from 0.145 to 8.76 (Table 5), with an average value of 2.97. The majority of these values are more than seawater (0.19) and less than rainwater (7.14) (Table 5). This is due to the presence of CaCO_3_ minerals (Aragonite, Calcite, and Dolomite) and Gypsum within the aquifer matrix, leading to CO_2_-CaCO_3_ and CO_2_-CaMg(CO_3_)_2_ interactions supported by weathering of gypsum deposits^29^.

#### (rSO42−/rCl−)

This ratio serves as a guide for detecting any excess sulfate in the groundwater (due to CaSO_4_ dissolution or CaCO_3_ precipitation) associated with sulfate mineral dissolution. The mean value of the (SO_4_^2−^/Cl^−^) ratio for the groundwater samples (Table 5) is 0.33. The solubility of sulfate mineral increases in the groundwater system by depth and distance from the recharge to the discharge area^38^. Consequently, the infiltration of meteoric water with the greater distance from the recharge location is taken into consideration as the principal factor for increasing the sulfate’s attention with the aid of using direct motion of leaching processes. The (SO_4_^2−^/Cl^−^) ratio of the majority (71%) of the groundwater samples in the study area has values greater than those in seawater (0.1), indicating the solution of terrestrial sources of sulfate such as gypsum and anhydrite.

### Mixing model

**Fig. 12:**
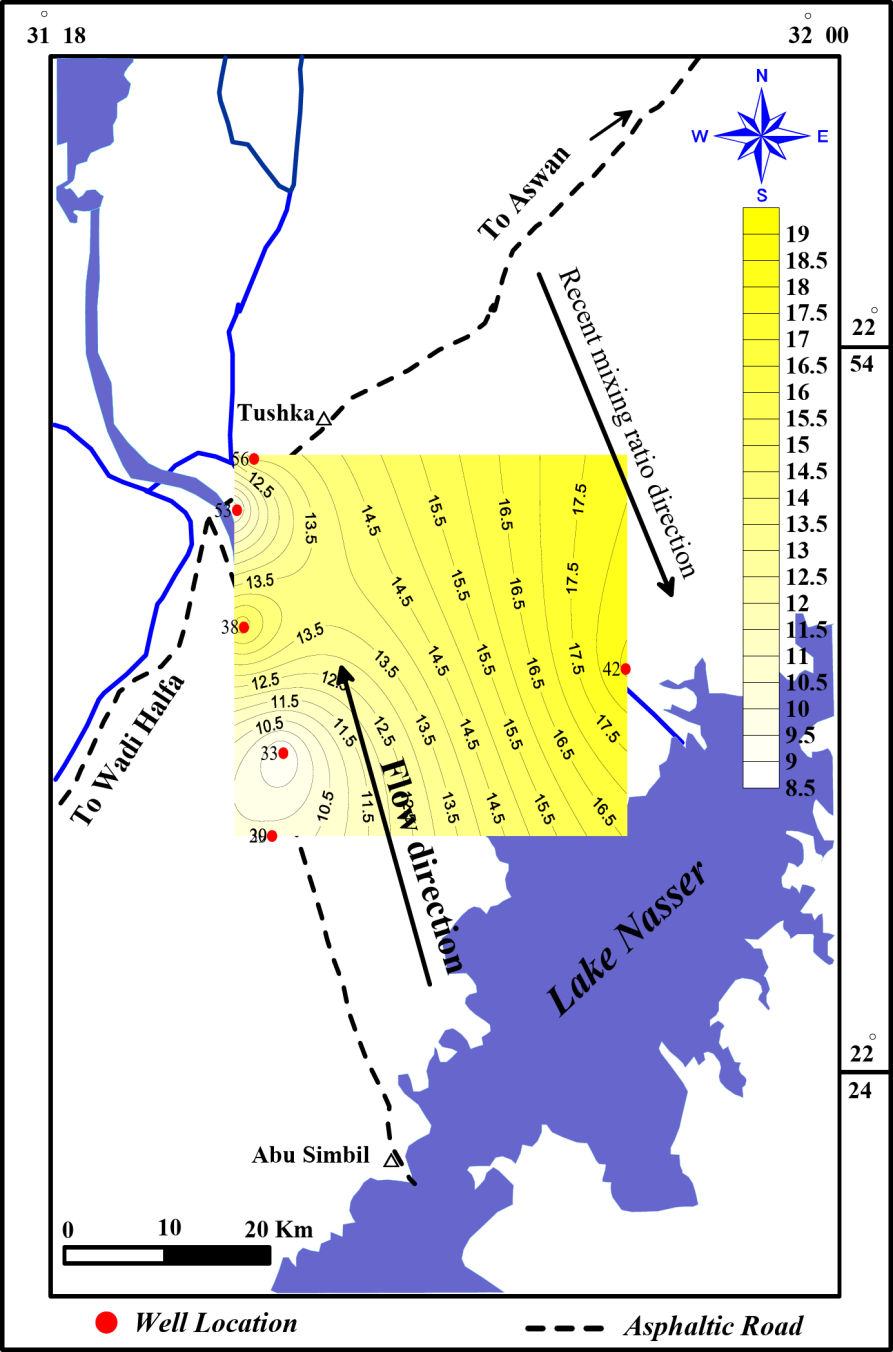
Mixing ratio contour map.

Globally, it has been observed that deep aquifers dominated by fossil water may contain a detectable level of recent contaminants, which points to recent meteoric recharge^39^. According to^24^ groundwater in the Nubian Sandstone Aquifer (NSSA) seems to be a mixture of recent meteoric water and paleo-groundwater. In the study area, groundwater from the water-bearing formation (Nubian sandstone) has paleowater^40^ contribution percent ranging from 81 to 92% and Lake asser water contribution percent ranging from 7 to 18% (Table 8) From the mixing ratio contour map, it is clear that the mixing ratio of the recent recharge increases in the southeast direction where the lake Nasser considers the opposite flow direction which indicates that the recent recharge comes from the lake Nasser water (Fig. 12).

**Table 8:** Groundwater isotopic data gathered from the study area.

Sample no.	δ^18^O (‰)	δ^2^H (‰)	Paleowater %	Lake Nasser water %	GMWL
23	− 10.1	− 79.2	92.707	7.293	
29	− 9.74	− 74.4	89.789	10.211	− 70.8
30	− 9.77	− 78.2	90.032	9.968	− 67.92
33	− 9.9	− 78.3	91.086	8.914	− 68.16
38	− 9.1	− 75.2	84.603	15.397	− 69.2
42	− 8.7	− 71.6	81.361	18.639	− 62.8
53	− 9.8	− 75.5	90.276	9.724	− 59.6
56	− 9.3	− 77.8	86.224	13.776	− 68.4
Lake Nasser	1.34	19.5			− 64.4
NSSA	− 11	− 80			20.72

**Fig. 13:**
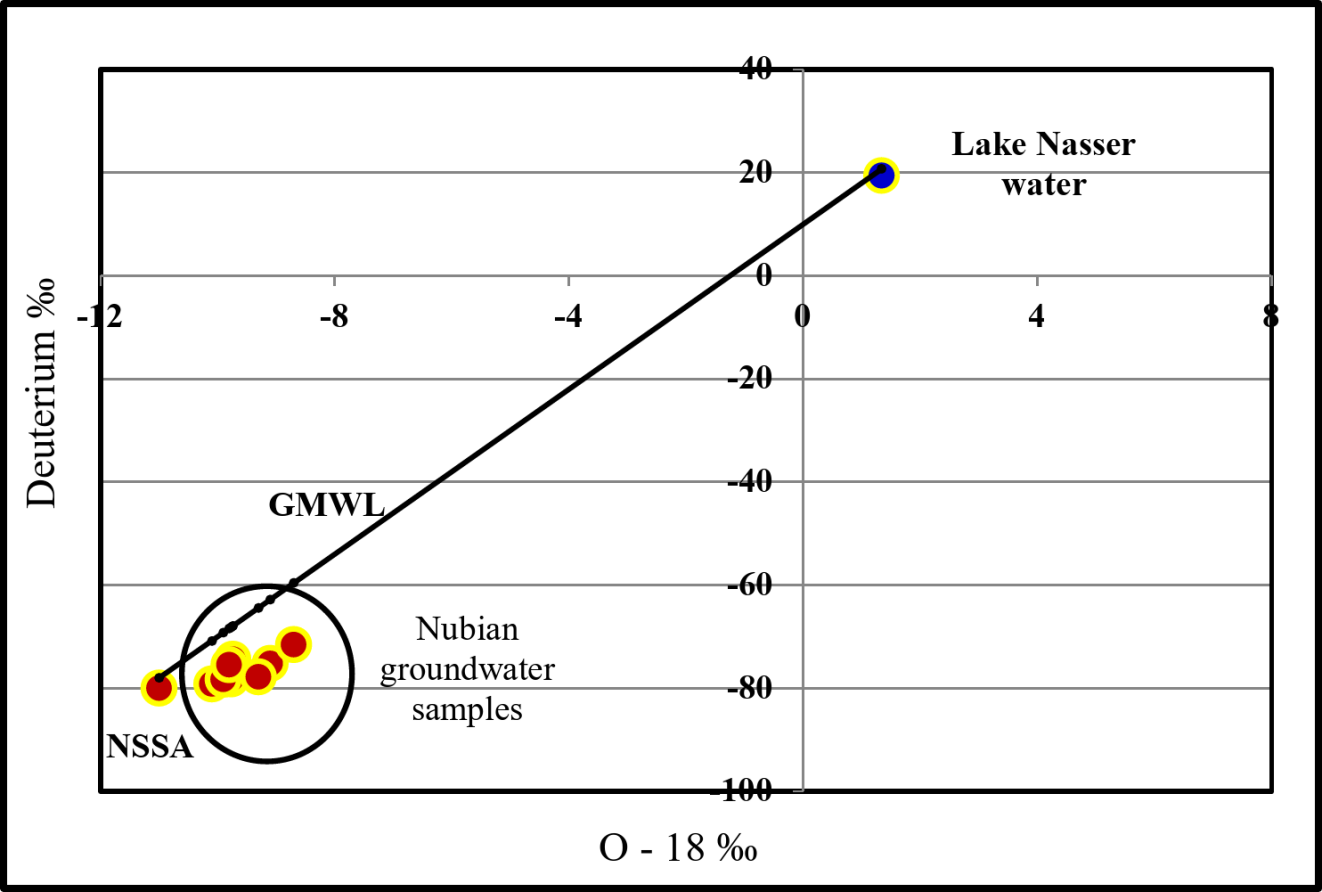
Relationship between oxygen-18 and deuterium contents.

Plots of δ 18O versus δ D showed that the groundwater of the study area is very much depleted in both 18O and D for all the water samples (inside the circle, Fig. 13). While the meteoric water isotopic composition of recent rain reaches δ D = δ 18O + 10 and Lake Nasser water δ 18O = 1.34 & δ D = + 19.5. All the groundwater samples fell under the global meteoric water line (GMWL= (8* δ 18O) + 10)^41^, indicating that they are mainly of meteoric origin.

### Extent of nitrate and heavy metals pollution

Table 9 presents a statistical summary of the laboratory results and their comparisons with recognized water quality standards. Nitrate concentrations are below the maximum allowable limits of the^42^. However, high concentrations of heavy metals were recorded in the groundwater samples (Table 2) at various extents. It was observed that 92%, 885, 64%, 12%, and 8% of the total water samples were contaminated with Al, Fe, Mn, Ni, and Cr, respectively. Furthermore about 4% of the total samples were contaminated with Cu and Pb (Table 9). The poor groundwater quality of the study area may be due to rapid urbanization, industrial effluents, mechanical and electronic waste, poor waste management, and hydrogeological setting^7^.

**Table 9:** Standard limits for drinking and irrigation water for the groundwater in the investigated area.

Parameters mg/l	Max. Min.	Average	Average acceptable (WHO, 2017)	No of samples	% Contaminated samples according to (WHO 2017)	Sample code numbers
Ba	0.17 0.02	0.05	0.7	25	0	–
B	N.D N.D	N.D	0. 5	25	0	–
Ag	N.D N.D	N.D	0.05:0.5	25	0	–
Pb	0.27 0.001	0.015	0.010	25	4	8
Mo	N.D N.D	N.D	–	25	0	–
Fe	25.9 0.04	8.95	0.3	25	88	2, 3, 4, 5, 6, 7, 8, 9, 10, 11, 12, 13, 14, 15, 16, 17, 18, 19, 20, 21, 22, 23
Cu	0.48 0.001	0.026	0.02	25	4	6
Mn	0.91 0.002	0.3	0.2	25	64	4, 6, 7, 8, 9, 10 ,11, 13, 14, 16, 17, 18, 19, 20, 21, 22,
Sr	0.95 0.07	0.28	–	25	0	–
Cd	N.D N.D	N.D	0.01	25	0	–
Cr	0.29 0.0023	0.025	0.05		8	5 and 6
Ni	0.78 0.0007	0.051	0.07	25	12	1, 2 and 4
NO_3_	29 1.4	8.71	50	25	–	–
Al	0.59 0.07	0.05	0.1	25	92	All samples except 3 and 5

## Conclusion

The Nubian sandstone aquifer is the main aquifer in the study area, where it is represented by 24 groundwater samples plus one sample from the stem of Lake Nasser. All samples were collected, analyzed, and interpreted. Groundwater salinity classification shows that all the groundwater samples are fresh water types except one sample considered brackish water type according to the Gattacceca classification. The relationship between salinity content and the other ions shows a strong correlation between the values of Ca^2+^, Cl^−^, and SO_4_^2−^ with the TDS values and a moderate correlation between Na^+^ values with TDS values. Silicate and carbonate weathering are the main hydrochemical processes affecting the groundwater. From the results of the mixing Model, it is indicated that the Nubian sandstone aquifer has a paleowater contribution percent ranging from 81 to 92% and Lake Nasser water contribution percent ranging from 7 to 18%. The nitrate concentrations are below the maximum allowable limits of the WHO. However, high concentrations of heavy metals were recorded in the groundwater samples at various extents.

## Data Availability

The datasets generated and analyses during the current study are available in full in the Manuscript.
